# Strategies to Recruit a Diverse Low-Income Population to Child Weight Management Programs From Primary Care Practices

**DOI:** 10.5888/pcd14.170301

**Published:** 2017-12-21

**Authors:** Sarah E. Barlow, Nancy F. Butte, Deanna M. Hoelscher, Meliha Salahuddin, Stephen J. Pont

**Affiliations:** 1 Texas Children’s Hospital, Baylor College of Medicine, Houston Texas; 2 USDA/ARS Children’s Nutrition Research Center, Department of Pediatrics, Baylor College of Medicine, Houston, Texas; 3 Michael & Susan Dell Center for Healthy Living, The University of Texas Health Science Center at Houston (UTHealth)–School of Public Health, Austin, Texas; 4 Texas Center for the Prevention and Treatment of Childhood Obesity, Dell Children’s Medical Center, University of Texas at Austin Dell Medical School, Austin, Texas

## Abstract

**Purpose and Objectives:**

Primary care practices can be used to engage children and families in weight management programs. The Texas Childhood Obesity Research Demonstration (TX CORD) study targeted patients at 12 primary care practices in diverse and low-income areas of Houston, Texas, and Austin, Texas for recruitment to a trial of weight management programs. This article describes recruitment strategies developed to benefit both families and health care practices and the modification of electronic health records (EHRs) to reflect recruitment outcomes.

**Intervention Approach:**

To facilitate family participation, materials and programs were provided in English and Spanish, and programs were conducted in convenient locations. To support health care practices, EHRs and print materials were provided to facilitate obesity recognition, screening, and study referral. We provided brief training for providers and their office staffs that covered screening patients for obesity, empathetic communication, obesity billing coding, and use of counseling materials.

**Evaluation Methods:**

We collected EHR data from 2012 through 2014, including demographics, weight, and height, for all patients aged 2 to 12 years who were seen in the 12 provider practices during the study’s recruitment phase. The data of patients with a body mass index (BMI) at or above the 85th percentile were compared with the same data for patients who were referred to the study and patients who enrolled in the study. We also examined reasons that patients referred to the study declined to participate.

**Results:**

Overall, 26% of 7,845 patients with a BMI at or above the 85th percentile were referred to the study, and 27% of referred patients enrolled. Enrollment among patients with a BMI at or above the 85th percentile was associated with being Hispanic and with more severe obesity than with patients of other races/ethnicities or less severe obesity, respectively. Among families of children aged 2 to 5 years who were referred, 20% enrolled, compared with 30% of families of older children (>5 y to 12 y). Referral rates varied widely among the 12 primary care practices, and referral rates were not associated with EHR modifications.

**Implications for Public Health:**

Engagement and recruitment strategies for enrolling families in primary care practice in weight management programs should be strengthened. Further study of factors associated with referral and enrollment, better systems for EHR tools, and data on provider and office adherence to study protocols should be examined. EHRs can track referral and enrollment to capture outcomes of recruitment efforts.

## Introduction

Obesity is a prevalent chronic condition in childhood with lifelong health consequences. Effective weight management programs for children must be behavior-based, comprehensive, and of moderate to high intensity to improve weight status in the immediate and intermediate term (1). However, such programs cannot improve obesity at a population level unless they are broadly disseminated and adopted, especially by those who are at greatest risk, which include Hispanic and black children (2), and because poverty is often associated with childhood obesity (3), children of limited financial means.

Development of effective dissemination and implementation strategies requires focus on the process of recruitment and engagement and the outcomes from those efforts (4). Primary care practices are important sites for weight management promotion because 75% of children aged 18 years or younger see primary care providers each year (5). Assessment of body mass index (BMI) and subsequent intervention to address the condition is recommended care, either in the provider’s office or by referral to an adjunct obesity management program. Methods through which patients in primary care practices are placed in weight management programs should be studied.

Limited studies have assessed recruitment and enrollment in childhood weight management programs through primary care practices. One systematic review of clinical trials of obesity interventions that targeted low-income or minority children presented limited recruitment and retention information from among the 38 studies reviewed that reported any recruitment information, including strategies, setting, duration, barriers, and effects (6). Four studies recruited participants from primary health care practices (though not exclusively), and 2 of these reported rates of enrollment after referral of 62.5% in an adolescent study and 22.2% in a preschool study (6–10). In a child weight management program in a multispecialty health care practice, 41% of patients referred to a weight management program attended an initial presentation (9). A few studies (10,11) compared rates of enrollment relative to potentially eligible patients, not only those who were physician-referred. One study of adolescents evaluated several strategies to refer patients, one of which bypassed the medical provider by sending letters to patients deemed qualifying from electronic health record (EHR) data; that study found that approximately 9% of patients with obesity enrolled (12). This rate was similar to one from a German study of a low-intensity telephone-based obesity intervention targeting patients with obesity (13). Because studies in the area of recruitment and retention in childhood obesity studies are limited, data about recruitment strategies and associated factors, especially in low-income populations, are needed to inform future programs. Reasons that patients or families reported for not enrolling in weight management programs and for program drop-out included inconvenient locations or time of programs and also lack of perceived need (13–15). Barriers that providers reported included low self-efficacy, perceived need for counseling and communication support, lack of reimbursement, and time constraints (16, 17).

The Texas Childhood Obesity Research Demonstration (TX CORD) study presented an opportunity to develop and evaluate recruitment strategies for weight management programs offered to a large population of a Medicaid-eligible, diverse sample of children seen in TX CORD primary care practices. This study describes the TX CORD recruitment strategy and study findings, taking advantage of the unusual availability of demographic and anthropometric information on a large but defined cohort of eligible participants.

## Purpose and Objectives

The TX CORD study was a multilevel, multisystem intervention to address childhood overweight and obesity in children aged 2 to 12 years from racially/ethnically diverse, low-income catchment areas in Houston, Texas, and Austin, Texas (18,19). The study examined recruitment to a 12-month randomized controlled trial (RCT) that was embedded in the population-level, systems-based intervention. Twelve Houston and Austin primary care practices received training and materials to optimize identification and care of all patients with overweight and obesity. For the RCT, patients aged 2 to 12 years from these practices with a BMI at or above the 85th percentile were recruited to a study that compared a community-based program, which used both “Mind, Exercise, Nutrition . . . Do It! (MEND) and an adapted Coordinated Approach to Child Health (MEND–CATCH) program, with a health care–based program that used the materials provided to the practices (Next Steps) (18). Participants were stratified into 3 age groups: 2 to 5 years, 6 to 8 years, and 9 to 12 years. The objectives of this study were to describe and evaluate the strategies used in primary care practices to recruit families of children with overweight or obesity into weight management programs.

Twelve partner primary care practices in Houston and Austin participated in the trial. The Houston practices were part of a large hospital organization with a single EHR system. Five Houston practices were selected because they were located in the TX CORD catchment area. Three of these were designated medical homes aimed at providing care to Medicaid and CHIP (Children’s Health Insurance Program)–eligible children, and the other two had 30% to 50% of patients covered by Medicaid or CHIP. The 7 partner practices in the Austin catchment area were federally qualified health centers and nonprofit safety-net primary care clinics. The Austin practices were members of 3 different health care organizations and used 3 different EHR systems.

All offices had 2 to 5 full-time equivalent pediatric providers, and some had social workers on staff. Their patients were generally low-income and nonwhite. Catchment areas included Hispanic and black neighborhoods (Houston) or mostly Hispanic neighborhoods (Austin). Study recruitment was limited to health care practices to ensure that children in weight management programs had a source of health care to identify and manage any physical or mental comorbidities. Partner sites were able to provide appropriately de-identified data from their EHRs to describe their clinic population.

## Intervention Approach

### TX CORD intervention

The TX CORD study implemented primary obesity prevention strategies at schools and early childhood education centers within the catchment areas in Houston and Austin. The TX CORD secondary obesity prevention study (aimed at children with BMI ≥85th percentile) was a 2-arm RCT that compared a community intervention and a health care intervention that took place within the primary prevention catchment areas. The interventions were 12 months. Five cohorts were enrolled from September 2012 through January 2014, and the last cohort ended in January 2015. Participants in both arms had data on height and weight, fitness level, dietary intake, and psychosocial factors measured at baseline, 3 months, and 12 months. Children and parents who were randomized to the community intervention arm first participated in a 3-month intensive program, MEND/CATCH, which was held at a YMCA facility in the catchment area. Families of preschool children (aged 2–5 y) attended weekly 90-minute sessions that focused on healthy food identification, parent–child games for physical activity, and parenting skills. Children aged 6 to 12 years and their parents attended twice-weekly sessions that consisted of 1 hour of nutrition and behavior change lessons and 1 hour of physical activity for the children while parents had further facilitator-led group discussion. During months 4 through 12, all age groups had monthly family review sessions, with cooking classes and narrative role models, and children aged 6 to 12 years transitioned to twice-weekly YMCA youth sports (20).

Children and parents randomized to the comparison health care intervention arm were asked to discuss weight and healthy lifestyle with the provider during clinic visits by using Next Steps counseling material and a self-paced workbook for parents and children (21). Providers were encouraged to use the Next Steps material with any clinical patient; therefore, the use of Next Steps was not limited to RCT participants. Rather, families enrolled in the RCT and randomized to this arm received usual care that had been optimized for the practices with the training and Next Steps material. Visit frequency was determined by the provider and family together and was influenced by Texas Medicaid policy, which does not reimburse for visits to primary care providers solely to treat obesity.

### Engagement process and recruitment strategies

**Figure 1:**
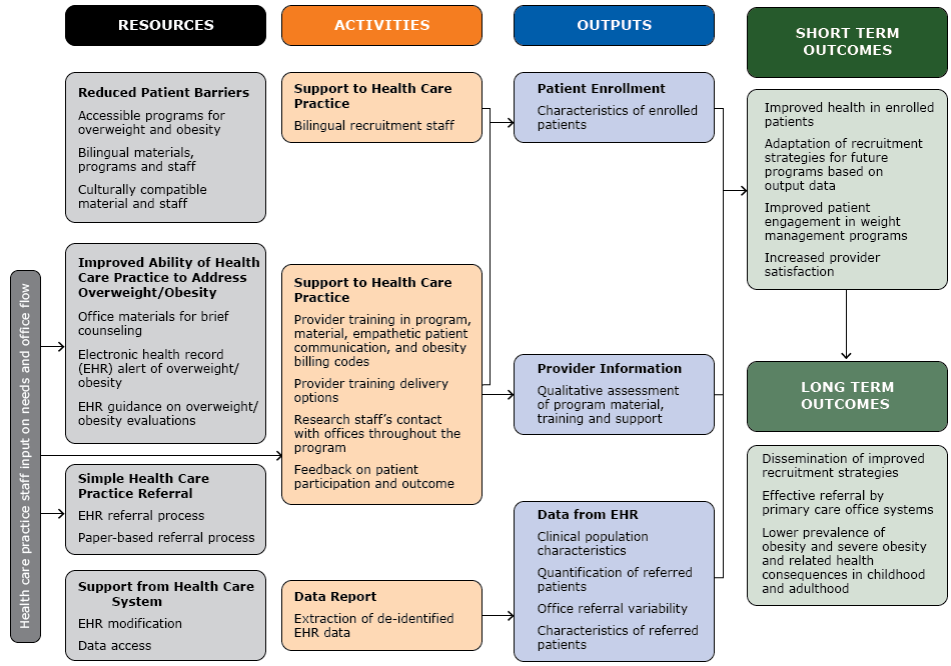
Framework to optimize recruitment of patients for the Texas Childhood Obesity Research Demonstration (TX CORD) study, Texas, 2012–2014.

Figure 1 presents the proposed framework of the TX CORD intervention. Resources and activities were guided by the perspectives of practices, and the referral process addressed the needs of both patients and practices. The framework consisted of defined resources, activities, and projected outputs and short- and long-term outcomes.

For the convenience of patients, the programs in the community arm of the RCT were scheduled for early evenings or Saturday mornings at YMCAs in catchment areas. To engage Spanish-speaking families, all program materials for both intervention arms of the RCT were in Spanish and English so that bilingual staff could guide families through the recruitment and consent process. Program material, which contained information such as food choices and meal routines, was culturally appropriate for participants. Team members who taught the community program at the YMCA were Hispanic and black.

The research staff met with providers and their staff members at each primary care practice to discuss the proposed study and to elicit concerns as the study protocol was developed and finalized. Providers identified time constraints and difficulty changing patient and family behaviors as challenges in addressing obesity. Obesity counseling competed with other anticipatory guidance during annual well-child examinations, and the cost of follow-up visits to address obesity alone was not covered under Medicaid or CHIP. Providers and their staff members wanted to ensure that clinical encounters were reimbursable and to preserve work flow when they provided weight counseling or made study referrals, and they had limited time for training. Materials and processes were finalized to respond to these concerns.

EHRs were modified to support obesity discussion and to increase referral to the study. On the basis of work done in a previous study (22), an alert was adapted for use in the EHR encounter when a patient aged 2 to 12 years had a BMI at or above the 85th percentile. The alert suggested, but did not require, use of a set of obesity-related diagnosis codes, laboratory orders, referrals, and education materials (ie, Next Steps). This EHR set included a process to indicate family permission to be contacted about the study. This approach was proposed by staff members of provider practices to minimize study-referral paperwork. However, all practices also had paper referral forms for faxing recruitment information. Regardless of referral method, providers and their staff members were asked to introduce the study only briefly; members of the research staff then called interested families to explain the study and to determine qualification.

Next Steps counseling materials were adapted to help providers introduce obesity and obesity prevention strategies to patient families. Such materials served as an engagement tool in study recruitment but were also resources for the health care arm of the RCT. Next Steps materials presented a list of healthy lifestyle themes displayed on a poster and in a desktop flip chart with simple graphics and counseling tips (21) Parents and providers identified 1 or 2 themes of greatest interest and relevance for the family to use in brief counseling during an office visit. By being visible in the examination room, the poster could also cue families to initiate a conversation about lifestyle even if the provider did not.

A 2-hour training was developed for providers that included orientation to the study, EHR modifications, and the Next Steps materials. It also included Texas Medicaid coding rules for the diagnosis of obesity and a brief introduction to motivational interviewing, with a goal of facilitating discussion with families about overweight and obesity and encouraging families to initiate change.

### Engagement implementation

Although the EHR modifications were planned for all offices, the Austin health care systems underwent several major administrative changes, which delayed EHR modifications until the study was completed. Therefore, Austin offices had no EHR flag for overweight or EHR support for obesity care, and study referral in Austin was exclusively via paper and fax.

The planning and training of practice staff members were conducted during spring and summer 2012. Providers (pediatricians, nurse practitioners, and social workers) received a 2-hour training in one of several different formats: in person, by live webinar, or by recorded webinar. Active recruitment and study enrollment began within weeks of the training. Briefly, providers identified eligible patients (eg, children with a BMI ≥85th percentile), assisted by the EHR alert. Providers used Next Steps materials and EHR support to address obesity and referred patients for recruitment.

Once interested families were referred, research staff members telephoned them to explain the study, assess eligibility, and offer an enrollment visit for consent, assent, measurement, and randomization. Three to 5 contact attempts were made. Outcome, including parents’ reasons for not enrolling in the study, were tracked. Because recruitment of children aged 2 to 5 years was difficult, a secondary recruitment process was initiated in which offices generated lists of recent encounters with eligible children aged 2 to 5 years (with a BMI ≥85th percentile). These families were then contacted by telephone from the practice, given a brief description of the program, and asked if research staff members could contact them.

Once enrolled, patients in the health care arm received the intervention self-paced booklet and encouragement to schedule additional provider visits, and the patients enrolled in the community program participated in the MEND/CATCH program described. Participants in the 12-month study were recruited and enrolled in 5 waves from September 2012 through January 2014.

To support practices, research staff members visited each practice every 2 or 3 months to remind the staff of the study, answer questions, and replace missing material. In addition, practices received information about outcomes for referred patients and also height, weight, and BMI measures at 3 and 12 months of those who participated in both the community and the health care arms of the study.

## Evaluation Methods

To understand the characteristics of the large but circumscribed patient population designated for recruitment, we used EHR data provided by the 12 partner offices to examine the demographic and anthropometric characteristics of all children aged 2 to 12 years seen during recruitment (September 2012 through January 2014). The following de-identified information was included: age; sex; race/ethnicity (Hispanic, non-Hispanic black, non-Hispanic white, and other); insurance type (Medicaid, CHIP, commercial or other, which included Tricare, Medical Access Program in Travis County, and unknown); and weight and height, which were used to calculate BMI and BMI percentile and to categorize children as overweight (BMI 85th to <95th percentile), obese (95th to <99th percentile), or severely obese (≥99th percentile). When patients had multiple encounters, the variables associated with the first well-child visit during the recruitment period were used. For patients without well-child visits, the first urgent encounter in which weight and height were measured was used, and when no height was obtained at any encounter, nonanthropometric variables from the first urgent visit were used. Differences between Houston and Austin cohort characteristics were examined. Prevalence of overweight and obesity was compared with NHANES (National Health and Nutrition Examination Survey) 2011–2012 data by age and race/ethnicity (2).

From these office data, the eligible population was defined as children with a BMI at or above the 85th percentile, and their demographic characteristics were examined. The referred patients were the families of the eligible children who agreed to be contacted by our research staff. Children’s data were limited to age, sex, weight, and height, and some measures were missing or were from parent report rather than provider report. The enrolled patients were families who consented to the study, and their children’s data came from baseline research evaluations, including measured weight and height and parent-reported race/ethnicity and insurance type. The demographic and anthropometric characteristics of the 3 groups (eligible population, referred patients, and enrolled patients) were compared to examine characteristics of children who were likely to progress to referral and enrollment.

By using the study database as well as information from the calls from our research staff to referred families, the outcomes of referred families were categorized into 1) enrollment into the study; 2) ineligibility because a medical or psychological condition made the community program unsuitable for the child; 3) ineligibility because of research criteria, which limited enrollment to one child per family and to domicile within 5 miles of the catchment area borders; 4) lack of transportation to the community program; or 5) lack of interest, which included families who actively declined and those who did not respond to multiple telephone calls. These outcomes were examined by age group. A questionnaire completed by provider practices at the end of the study provided perspective on study training and participation. To examine variation in office engagement implementation strategies, the proportions of patients referred and enrolled were examined by office, and association between availability of EHR tools and patient referral and enrollment was tested.

We used χ^2^ tests, univariate linear regression, and univariate logistic regression — for categorical, continuous, and binary variables, respectively — to evaluate the practice cohort, testing within age groups for differences between sites and by BMI status. The χ^2^ test was used to test differences in reasons for nonenrollment. A multivariable logistic regression model was used to calculate odds of enrollment of office patients with a BMI at or above the 85th percentile relative to nonenrollment within each age group and of referred patients relative to nonenrollment for each age group. The variables included in the multivariable logistic regression models were mutually adjusted for one another.

Institutional review boards for human subject research for The University of Texas Health Science Center, Baylor College of Medicine, and Seton Health Care Family approved the protocol.

## Results

### Health care office patient population in Houston and Austin

**Figure 2:**
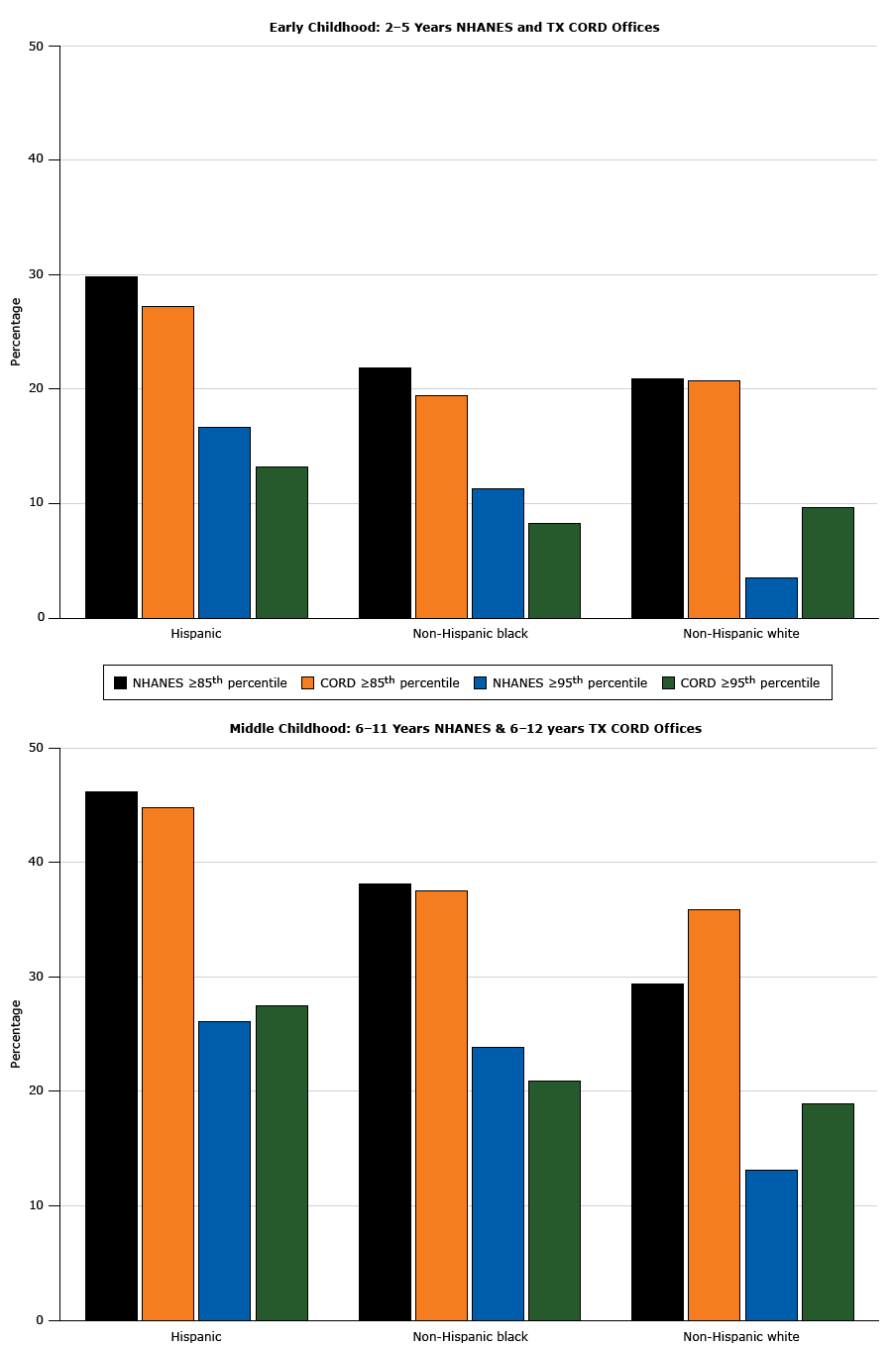
Prevalence of overweight and obesity among patients with a body mass index at or above the 85th percentile (N = 7,845) seen in Texas Childhood Obesity Research Demonstration (TX CORD) study practices, by racial/ethnic groups. Data are from NHANES 2011–2012 (2) and from participating TX CORD practices, 2012–2014. Abbreviations: NHANES, National Health and Nutrition Examination Survey. **Table:** 

Patients aged 2 to 12 years seen in the 12 participating primary care practices during the recruitment period were approximately 60% Hispanic patients and 20% non-Hispanic black patients across ages and sites. Approximately 70% of the patients were insured by Medicaid or CHIP. Table 1 presents the characteristics divided by age group and site. The proportion of patients with overweight or obesity (BMI ≥85th percentile) and obesity (BMI ≥95th percentile) in each racial/ethnic category and age group were similar to the national prevalence rates of NHANES 2011–2012 (Figure 2) (2). Houston practices had more patients than Austin practices, a higher percentage of non-Hispanic black patients, and a higher percentage of patients with commercial insurance. Although the distribution of BMI categories was different between the 2 sites in the 6-to-8 years age group and in the 9-to-12 years group, the proportions of children with obesity or severe obesity did not differ by site. The ages differed between sites, but the means were within 3 months of each other.

**Table 1:** Characteristics of Patients Aged 2 to 12 Years Seen in Primary Care Practices Participating in the Texas Childhood Obesity Research Demonstration Study (TX CORD), by Age Group and by Site, 2012–2014

Characteristic^a^	2–5 Years			6–8 Years			9–12 Years		
	Houston, n = 9,448	Austin, n = 3,707	*P* Value^b^	Houston, n = 4,707	Austin, n = 2,030	*P* Value^b^	Houston, n = 4,666	Austin, n = 2,202	*P* Value^b^
**Age, mean (SD), y **	3.6 (1.2)	3.5 (1.1)	0.007	7.3 (0.9)	7.1 (0.9)	<.001	10.8 (1.2)	10.9 (1.3)	.22
**Sex**									
Female	4,691 (49.7)	1,856 (50.1)	.67	2,288 (48.6)	993 (48.9)	.84	2,261 (48.5)	1,099 (49.9)	.26
Male	4,757 (50.3)	1,851 (49.9)		2,416 (51.4)	1,037 (51.1)		2,405 (51.5)	1,103 (50.1)	
**Race/ethnicity**									
Hispanic	5,229 (57.8)	2,369 (66.6)	<.001	2,622 (58.9)	1,267 (65.3)	<.001	2,540 (56.8)	1,670 (79.1)	<.001
Non-Hispanic black	2,374 (26.3)	232 (6.5)		1,177 (26.4)	149 (7.7)		1,314 (29.4)	147 (7.0)	
Non-Hispanic white/other	1,436 (15.9)	954 (26.8)		656 (14.7)	523 (27.0)		616 (13.8)	293 (13.9)	
**Insurance**									
Medicaid	5,472 (59.0)	3,212 (86.8)	<.001	2,514 (54.8)	1,537 (75.9)	<.001	2,308 (51.3)	1,319 (60.1)	<.001
CHIP	640 (6.9)	246 (6.6)		615 (13.4)	248 (12.2)		635 (14.1)	288 (13.1)	
Commercial	3,104 (33.5)	156 (4.2)		1,438 (31.3)	74 (3.7)		1,533 (34.1)	150 (6.8)	
Other	62 (0.7)	86 (2.3)		20 (0.4)	166 (8.2)		20 (0.4)	438 (20.0)	
**BMI percentile, mean (SD) **	55.2 (31.6)	54.4 (32.3)	.20	67.1 (29.0)	63.3 (31.2)	<.001	70.1 (29.4)	70.6 (29.5)	.52
**BMI percentile category**									
<5th	627 (7.5)	310 (8.5)	.28	105 (2.7)	93 (4.7)	.001	100 (2.5)	66 (3.0)	<.001
5th to <85th	5,733 (68.8)	2,465 (67.4)		2,350 (59.9)	1,210 (60.6)		2,068 (51.8)	1,102 (50.7)	
85th to <95th	1,048 (12.6)	460 (12.6)		604 (15.4)	292 (14.6)		734 (18.4)	418 (19.2)	
95th to <99th	533 (6.4)	255 (7.0)		587 (15.0)	279 (14.0)		823 (20.6)	499 (23.0)	
≥99th	392 (4.7)	168 (4.6)		275 (7.0)	123 (6.2)		267 (6.7)	88 (4.0)	

Abbreviations: BMI, body mass index; CHIP, Children’s Health Insurance Program; SD, standard deviation.

a Values are n (%) unless otherwise indicated. Denominators vary because the number of participants who responded to individual questions varied.

b *P* Values calculated by univariate linear regression tests, univariate logistic regression tests, and χ^2^ tests for continuous, binary, and categorical variables, respectively.

Age Category	Percentile by Data Source	Hispanic, %	Non-Hispanic Black, %	Non-Hispanic White, %
Early childhood (aged 2–5 y)	NHANES ≥85th	29.8	21.9	20.9
	TX CORD ≥85th	27.2	19.4	20.7
	NHANES ≥95th	16.7	11.3	3.5
	TX CORD ≥ 95th	13.2	8.3	9.7
Middle childhood (aged 6–12 y)	NHANES ≥ 85th	46.2	38.1	29.4
	TX CORD ≥ 85th	44.8	37.5	35.9
	NHANES ≥ 95th	26.1	23.8	13.1
	TX CORD ≥ 95th	27.5	20.9	18.9

When the data were examined by healthy weight (BMI <85th percentile) versus overweight/obesity (≥85th percentile) in each age group (Table 2), those with overweight or obesity more often were Hispanic and had Medicaid or CHIP rather than commercial insurance.

**Table 2:** Characteristics of Patients Aged 2 to 12 Years Seen in Primary Care Practices Participating in the Texas Childhood Obesity Research Demonstration Study, by Age Group and BMI Status^a^, 2012–2014

Characteristic^b^	Office								
	2-5 Years			6-8 Years			9-12 Years		
	<85, n = 9,135	≥85, n = 2,856	*P* c Value	<85, n = 3,758	≥85, n = 2,160	*P* c Value	<85, n = 3,335	≥85, n = 2,829	*P* c Value
**Age y, mean (SD) **	3.5 (1.21)	3.9 (1.18)	<.001	7.2 (0.90)	7.4 (0.91)	<.001	10.8 (1.21)	10.9 (1.2)	.05
**Sex**									
Female	4,554 (49.9)	1,384 (48.5)	.12	1,869 (49.7)	1,027 (47.5)	.11	1,692 (50.7)	1354 (47.9)	.03
Male	4,581 (50.2)	1,472 (51.5)		1,889 (50.3)	1,133 (52.5)		1,643 (49.3)	1,475 (52.1)	
**Race/ethnicity**									
Hispanic	5,097 (58.3)	1,902 (68.9)	<.001	2,086 (58.6)	1,376 (66.3)	<.001	1,942 (61.0)	1,896 (69.4)	<.001
Non-Hispanic black	1,889 (21.6)	455 (16.5)		760 (21.4)	377 (18.2)		757 (23.8)	532 (19.5)	
Non-Hispanic white/other	1,756 (20.1)	402 (14.6)		713 (20.0)	321 (15.5)		484 (15.2)	304 (11.1)	
**Insurance**									
Medicaid	5,848 (67.6)	1,927 (72.0)	<.001	2,228 (62.8)	1,339 (66.2)	.006	1,728 (55.8)	1,489 (57.1)	<.001
CHIP	622 (7.2)	201 (7.5)		475 (13.4)	284 (14.0)		413 (13.3)	421 (16.1)	
Commercial	2,070 (23.9)	513 (19.2)		722 (20.3)	346 (17.1)		713 (23.0)	490 (18.8)	
Other	105 (1.2)	34 (1.3)		123 (3.5)	54 (2.7)		244 (7.9)	208 (8.0)	

Abbreviations: BMI, body mass index; CHIP, Children’s Health Insurance Program; SD, standard deviation.

a Healthy weight (<85th percentile) versus overweight or obese (≥85th percentile).

b Values are n (%) unless otherwise indicated.

c *P* values calculated by univariate linear regression tests, univariate logistic regression tests, and χ^2^ tests for continuous, binary, and categorical variables, respectively.

### Characteristics of eligible population, referred patients and enrolled patients

Of the 7,845 children with overweight or obesity seen in the TX CORD practices (Table 2), 2,030 (25.9%) were referred to the study, and 549 (27.0%) of those referred were enrolled (Table 3). Referral rates were lowest in the 9-to-12 year age group (22.7%), although the 28.7% referral rate among the 2 to 5 year age group reflects additional recruitment efforts implemented in this group because of low enrollment. Once referred, 32% of families with children aged 6-to-12 years enrolled, in contrast to 19.5% of those with children aged 2 to 5 years. Compared with the eligible population, the referred patients had more severe obesity, as assessed by both mean BMI and BMI category distribution. Severe obesity was present in 40% of age 2-to-5 years referrals, 36% of age 6-to-8 years referrals, and 26% of age 9-to-12 years referrals. Austin practices were smaller, making up 31% (aged 2–5 y), 32% (6–8 y), and 36% (9–12 y) of patients with BMI at or above the 85th percentile in the practices, yet the Austin practices accounted for larger proportions of the referred groups (38%, 38%, and 40%, respectively) and even larger proportions of the enrolled groups (52%, 54%, and 45%, respectively) than Houston offices. These data indicate higher referral and enrollment rates of their high BMI patients despite lacking the EHR referral tool. The enrolled patients had approximately the same levels of obesity as the referred groups. Although data on race/ethnicity and insurance were unavailable for referred patients, the proportion of non-Hispanic white patients was much lower among the enrolled than among the eligible population, and Medicaid enrollment was higher for enrolled patients than for the eligible population in the middle and youngest age groups.

**Table 3:** Sociodemographic and Anthropometric Characteristics of Patients With Overweight or Obesity (BMI ≥85th Percentile) Seen in Primary Care Practices Participating in the Texas Childhood Obesity Research Demonstration (TX CORD) Study, by Age Group^a^, 2012–2014

Characteristic	2–5 Years			6–8 Years			9–12 Years		
	Total^a^ (n = 2,856)	Referred (n = 822), 28.7	Enrolled (n = 160), 19.5	Total^a^ (n = 2,160)	Referred^a^ (n = 567), 26.3	Enrolled (n = 181), 31.9	Total^a^ (n = 2,829)	Referred (n = 641), 22.7	Enrolled (n = 208), 32.4
**Site, n (%)**									
Houston	1,973 (69.1)	508 (61.8)	83 (51.9)	1,466 (67.9)	352 (62.1)	97 (53.6)	1,824 (64.5)	385 (60.1)	93 (44.7)
Austin	883 (30.9)	314 (38.2)	77 (48.1)	694 (32.1)	215 (37.9)	84 (46.4)	1,005 (35.5)	256 (39.9)	115 (55.3)
**Age, mean (SD), y**	3.9 (1.18)	3.9 (1.04)	4.3 (1.02)	7.4 (0.91)	7.2 (0.88)	7.5 (0.85)	10.9 (1.2)	10.4 (1.05)	10.5 (1.04)
**Sex, n (%)**									
Female	1,384 (48.5)	426 (52.0)	84 (52.5)	1,027 (47.5)	281 (49.8)	84 (46.4)	1,354 (47.9)	291 (45.8)	104 (50.0)
Male	1,472 (51.5)	394 (48.0)	76 (47.5)	1,133 (52.5)	283 (50.2)	97 (53.6)	1,475 (52.1)	345 (54.2)	104 (50.0)
**Race/ethnicity, n (%)**									
Hispanic	1,902 (68.9)	NA	141 (88.1)	1,376 (66.3)	NA	153 (84.5)	1,896 (69.4)	NA	179 (86.1)
Non-Hispanic black	455 (16.5)		16 (10.0)	377 (18.2)		27 (14.9)	532 (19.5)		25 (12.0)
Non-Hispanic white/other	402 (14.6)		3 (1.9)	321 (15.5)		1 (0.6)	304 (11.1)		4 (1.9)
**Insurance, n (%)**									
Medicaid	1,927 (72.0)	NA	120 (82.2)	1,339 (66.2)	NA	116 (73.0)	1,489 (57.1)	NA	106 (58.2)
CHIP	201 (7.5)		12 (8.2)	284 (14.0)		24 (15.1)	421 (16.1)		38 (20.9)
Commercial	513 (19.2)		12 (8.2)	346 (17.1)		17 (10.7)	490 (18.8)		20 (11.0)
Other	34 (1.3)		2 (1.3)	54 (2.7)		2 (1.3)	208 (8.0)		18 (9.9)
**BMI percentile, mean (SD)**	93.9 (4.7)	96.5 (3.8)	97.0 (3.8)	94.9 (4.4)	97.0 (3.2)	97.3 (3.0)	95.0 (4.0)	97.0 (2.8)	97.3 (2.6)
**BMI percentile category**									
85th to <95th	1,508 (52.8)	127 (25.6)	37 (23.1)	896 (41.5)	98 (19.4)	35 (19.3)	1,152 (40.7)	99 (16.8)	29 (13.9)
95th to ≤99th	788 (27.6)	170 (34.2)	49 (30.6)	865 (40.1)	223 (44.2)	80 (44.2)	1,322 (46.7)	339 (57.4)	129 (62.0)
≥99th	561 (19.6)	200 (40.2)	74 (46.3)	399 (18.5)	184 (36.4)	66 (36.5)	355 (12.6)	153 (25.9)	50 (24.1)

Abbreviations: BMI, body mass index; CHIP, Children’s Health Insurance Program; NA, not applicable; SD, standard deviation.

a Total is the number of patients seen in the 12 participating TX CORD practices in each age group with a BMI at or above the 85th percentile. Referred is the number and percentage of patients with a BMI at or above the 85th percentile who were referred to the TX CORD study. Enrolled is number and percentage of patients referred to the study who enrolled.

**Figure 3:**
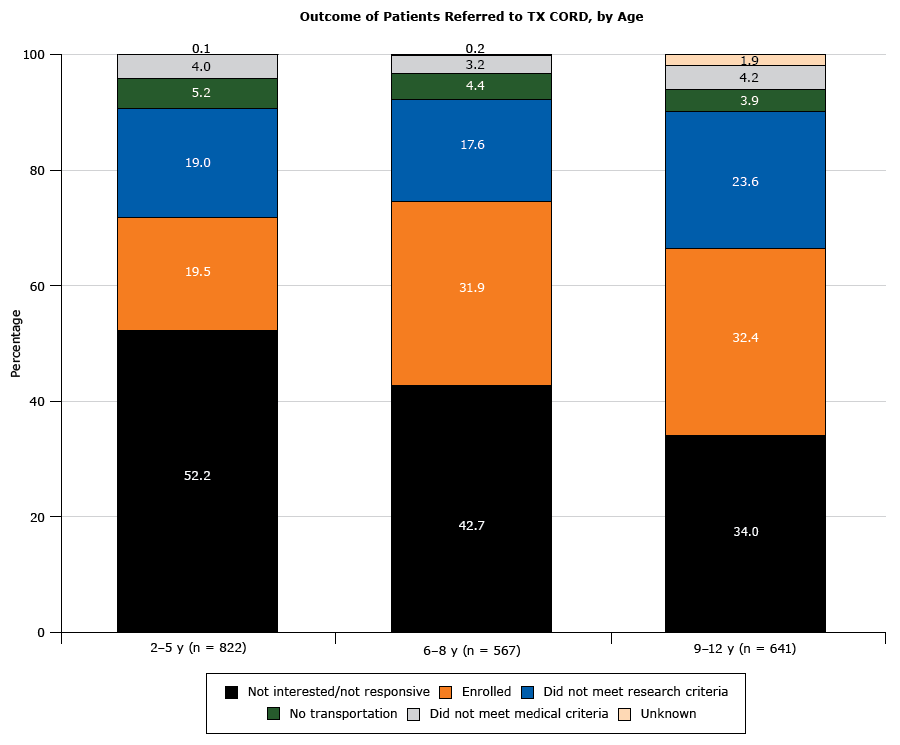
Outcome of patients with a body mass index at or above the 85th percentile (N = 2,030) referred to the Texas Childhood Obesity Research Demonstration (TX CORD) study. Among patients referred to the study, eligibility and interest varied by age group. **Table:** 

Multivariate analyses of variables associated with enrollment were performed for both the eligible population with BMI at or above the 85th percentile and the referred cohort. Enrollment for the eligible population was associated with more severe obesity, being from Austin, and being Hispanic or non-Hispanic black (Table 4). Sex and insurance categories were not associated with enrollment in any age group. For the referred cohort, higher BMI was not significantly associated with enrollment (Table 5). Significant predictors varied with age group; higher mean age within the age 2-to-5 years group, being from Austin within the age 9-to-12 years group, and both higher mean age and being from Austin within the age 6-to-8 years group (Table 5). The 3 patient age groups had different outcomes after referral (Figure 3). The age 2-to-5 years group had the highest proportion not interested and the lowest enrollment rate. The age 9-to-12 years group had the lowest proportion not interested and the highest proportion not meeting research criteria compared with the other age groups.

**Table 4:** Multivariate Analysis of Characteristics of Enrolled Patients (N = 549) Versus Eligible Patients (N = 7,531)in the Texas Childhood Obesity Research Demonstration (TX CORD) Study, by Age Group^a^, 2012–2014

Characteristic	2–5 Years (n = 2,725)		6–8 Years (n = 2,103)		9–12 Years (n = 2,703)	
	OR (95% CI)	*P* Value^b^	OR (95% CI)	*P* Value^b^	OR (95% CI)	*P* Value^b^
**Intercept**	0	NA	0	NA	0.05 (0.01–0.27)	NA
**Site**						
Houston	1 [Reference]					
Austin	2.61 (1.79–3.80)	<.001	2.89 (2.00–4.17)	<.001	2.86 (2.00–4.10)	<.001
**Sex**						
Male	1 [Reference]					
Female	0.98 (0.69–1.40)	.93	1.54 (1.09–2.16)	0.01	1.06 (0.77–1.44)	.73
**Age, y (continuous variable)**	1.45 (1.24–1.70)	<.001	1.33 (1.10–1.61)	.004	0.77 (0.68–0.88)	<.001
**Child race/ethnicity**						
Non-Hispanic white	1 [Reference]					
Hispanic	13.08 (4.08–41.89)	<.001	42.92 (5.94–310.12)	<.001	6.51 (2.37–17.86)	<.001
Non-Hispanic black	7.51 (2.08–27.11)	.002	28.72 (3.79–217.79)	.001	3.27 (1.07–10.03)	.04
**Insurance**						
Medicaid	1 [Reference]					
CHIP	1.05 (0.55–1.98)	.88	1.11 (0.69–1.79)	.66	1.20 (0.80–1.79)	.38
Commercial	0.64 (0.34–1.22)	.17	1.09 (0.62–1.93)	.76	1.15 (0.67–1.96)	.61
Other	0.91 (0.20–4.03)	.90	0.33 (0.08–1.45)	.14	0.83 (0.48–1.45)	.51
**BMI percentile category**						
85th to ≤95th	1 [Reference]					
95th to ≤99th	2.28 (1.44–3.62)	<.001	2.07 (1.34–3.20)	.001	3.39 (2.21–5.21)	<.001
>99th	4.91 (3.19–7.55)	<.001	3.95 (2.48–6.28)	<.001	5.12 (3.07–8.53)	<.001

Abbreviations: BMI, body mass index; CI, confidence interval; NA, not applicable; OR, odds ratio.

a Odds ratios of enrollment for eligible population (patients with BMI ≥85th percentile).

b *P* values calculated by multivariable logistic regression tests.

**Table 5:** Multivariate Analysis of Characteristics of Enrolled Patients (N = 1,589) Versus Referred Patients (n = 549), Texas Childhood Obesity Research Demonstration (TX CORD) Study, by Age Group^a^, 2012–2014

Characteristic	2–5 Years (n = 496)		6–8 Years (n = 504)		9–12 Years (n = 589)	
	OR (95% CI)	*P* Value^b^	OR (95% CI)	*P* Value^b^	OR (95% CI)	*P* Value^b^
**Intercept**	0.06 (0.02–0.14)	NA	0.01 (0–0.04)	NA	0.07 (0.01–0.43)	NA
**Site**						
Houston	1 [Reference]					
Austin	1.36 (0.92–2.01)	.12	1.69 (1.15–2.48)	.008	2.24 (1.57–3.19)	<.001
**Sex**						
Male	1 [Reference]					
Female	0.85 (0.58–1.26)	.43	1.27 (0.87–1.86)	.21	1.25 (0.88–1.77)	.21
**Age, y (continuous variable)**	1.64 (1.35–2.00)	<.001	1.74 (1.38–2.19)	<.001	1.13 (0.96–1.33)	.15
**BMI percentile category**						
85th–95th	1 [Reference]					
95th–99th	0.86 (0.51–1.45)	.57	0.80 (0.48–1.35)	.40	1.44 (0.88–2.38)	.15
>99th	1.31 (0.80–2.16)	.28	1.05 (0.62–1.79)	.85	1.29 (0.73–2.26)	.38

Abbreviations: BMI, body mass index; CI, confidence interval; NA, not applicable; OR, odds ratio.

a Odds ratios of enrollment for referred patients.

b *P* values calculated by multivariable logistic regression tests.

Outcome	Age Group, %		
	2–5 y, n = 822	6–8 y, n = 567	9–12 y, n = 641
Not interested/not responsive	52.2	42.7	34.0
Enrolled	19.5	31.9	32.4
Did not meet research criteria	19.0	17.6	23.6
No transportation	5.2	4.4	3.9
Did not meet medical criteria	4.0	3.2	4.2
Unknown	0.1	0.2	1.9

**Figure 4:**
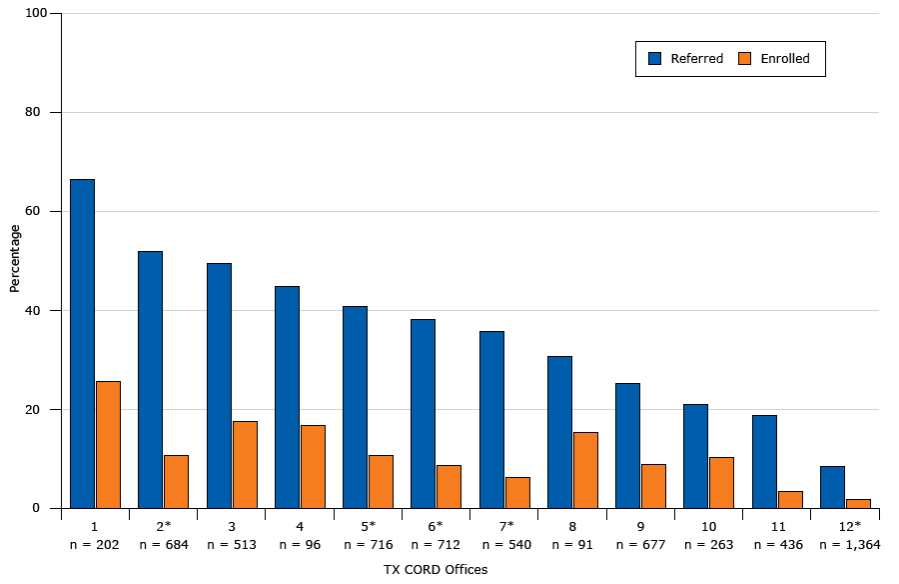
Percentage of patients in 12 primary care practices with a body mass index at or above the 85th percentile referred (N = 2,030) and enrolled (N = 549) in the Texas Childhood Obesity Demonstration (TX CORD) study, by primary care practice. Asterisks indicate that electronic health records for that office were modified to include a referral process for overweight or obesity. Numbers in parentheses are the total number of eligible patients in each practice. **Table:** 

The individual primary care practices varied considerably in referral and enrollment rates (Figure 4). Referral rates ranged from 8.5% to 66.8%. Enrollment ranged from 1.9% to 25.7% of the eligible population, and 17% to 50% of the referred population. Referral rates did not differ by availability of EHR referral tools.

Practice	No. Eligible Patients	% Eligible Patients Referred	Eligible Patients Enrolled
1	202	66.8	25.7
2*	684	52.0	10.8
3	513	49.5	17.7
4	96	44.8	16.7
5*	716	40.9	10.8
6*	712	38.2	8.7
7*	540	35.9	6.3
8	91	30.8	15.4
9	677	25.3	9.0
10	263	21.3	10.3
11	436	18.8	3.4
12*	1,364	8.5	1.9

The training and participation questionnaire, completed by 34 providers, offered 5 response options ranging from strongly agree to strongly disagree. Based on responses of agree or strongly agree, 50% reported that training provided adequate information, and 56% found the required time commitment acceptable. As a result of training and materials, 62% reported they were more likely to start a conversation about obesity as part of a patient encounter, and 65% felt more comfortable discussing obesity with families.

## Implications for Public Health

Primary health care practices are well positioned to identify children with overweight or obesity and address the problem through screening, counseling, and referral to a program, potentially improving dissemination and adoption of behavior-based weight management programs. The TX CORD RCT focused on primary health care practices for engagement and recruitment of diverse and low-income children at high risk for obesity to weight management programs. The implementation process had family-centered strategies with convenient time and location of programs and with program and recruitment elements that were welcoming to a predominantly Hispanic population. The process also had office-centered strategies, adding support to health care practices for recognizing and discussing unhealthy weight with patients and simple ways to refer them to support programs, including changes made to EHRs in 5 of the 12 offices. Because program participation was available only to those children in specific health care practices and because all the practices could provide limited, de-identified information from the EHR, this study had the capability of examining the pattern of referral and enrollment among a defined cohort.

In our study, recruitment began with an office visit that included screening and brief counseling for children who had overweight and obesity. The next step after screening was referral, and the final step was enrollment. Referral status reflects the activity of both the provider, who discussed the child’s weight and proposed referral, and of the family, which accepted referral. Thus, the recruitment strategies targeting family and primary care practice are relevant. Enrollment status after referral reflects a family decision based on perceived severity and individual circumstances, so office recruitment strategies may be less influential.

Approximately one quarter of the eligible population (patients aged 2 to 12 years with BMI ≥85th percentile seen in the health care offices) were referred. Referred patients were characterized by high body weight: about 40% of referred patients had severe obesity. Although the program targeted all patients at or above the 85th percentile, the pattern is consistent with experiences of hospital-based tertiary care weight management programs, in which most the patients are in the highest BMI category (21). Parent recognition of overweight and obesity increases with obesity severity (22), and, although not measured in this study, provider concern likely increases as well. Both factors could contribute to this referral pattern in TX CORD. Engagement of children with milder obesity is important and may lead to more success from the intervention (23). Although an important family-level engagement strategy was cultural compatibility with Hispanic families, we examined the effect only on enrolled families because referred families did not report race or ethnicity.

The high variation in referral rates across practices was unexpected. Training, support, and materials, with the exception of the EHR changes, were the same for all 12 offices. Providers reported moderate endorsement of the adequacy of training for the study and of the acceptability of the time commitment. Overall, providers agreed that the TX CORD experience led to more frequent discussion of obesity and more comfort with discussion. The EHR alert for BMI at or above the 85th percentile was designed to ensure provider recognition, but practices with the alert did not have higher referral rates, a finding that suggests that the EHR alert might need further development (eg, optimizing its location within the encounter template, ensuring a provider response). Austin practices as a group referred a higher percentage of eligible patients than Houston practices, but range of referrals rates in Austin offices (19% to 67%) and Houston offices (9% to 52%) were both wide. This variation may reflect differences in procedures among individual providers, given that the 12 participating practices had a low number of providers, but data on individual providers were not available. It is possible that providers needed more robust cues to action for referral. We did not audit providers or observe patient encounters, so future studies might consider more objective data on implementation of referral processes. The variation found in this study suggests that improvement is possible at the practice level, and exploration of office culture, office processes, and provider behavior may lead to interventions that support higher and more consistent referral rates across practices.

Enrollment, a family-level decision potentially influenced by interactions between provider and patient during the office visit, differed by race/ethnicity and age group. Hispanic patients, even after controlling for city and weight status, had a higher likelihood of enrollment. There have not been large studies of Hispanic children in weight management programs, and the culturally welcoming approaches that the study took with this group were effective. The proportion of enrolled patients was lower among children aged 2 to 5 years (20%) than among those aged 6 to 12 years (above 30%), although we used additional strategies to increase referral rates in this preschool group in response to low enrollment. The referral outcome data demonstrate the low interest and low response rate in the age 2 to 5 years group. Reasons could include less parental concern about weight in this age group (22) and more difficulty with logistics of attending the program. We did not see the expected association of higher enrollment with more severe obesity in the multivariate analysis of referred patients. Although high BMI increased likelihood of referral, decision to enroll may have been influenced by ability to participate in programs rather than by concern about obesity. Another explanation may be that social desirability led some families to accept referral from a concerned provider and then decline enrollment (actively or passively) because of low concern or other barriers. Future studies can note active refusal, but when programs cannot reach families, passive refusal cannot be distinguished from logistical challenges in low-income populations, such as cellular telephone inactivation.

Strengths of the study were the ability through use of EHR data to describe the large cohort of children with high BMI who were targeted for recruitment but did not enroll, and the implementation of the study in active, nonacademic practices. A limitation was the RCT structure, which could have influenced referral and enrollment. The referred group lacked race/ethnicity and insurance information, and lacked confirmation of the parent-reported weights and heights. In addition, recorded reasons for nonenrollment did not distinguish between active refusal and lack of response to contact efforts.

In conclusion, this study used a defined and well-characterized population of children at high risk for obesity and examined program recruitment and enrollment by using engagement and recruitment strategies that incorporated screening and brief counseling in primary care. The study successfully enrolled Hispanic families, but engagement of young children and children with less severe obesity was low. Providers reported increased obesity discussion during encounters as a result of the study, but referral varied widely by office. This variation suggests that low-referring offices could modify practices to increase attention to overweight and obesity among children, and focused study of high- and low-referring practices would be the next step in in developing interventions to address childhood obesity. Such interventions should include examination of EHR tools in actual clinical practice and further qualitative work to optimize their use.
